# Mechanistic Effects of E-Liquids on Biofilm Formation and Growth of Oral Commensal Streptococcal Communities: Effect of Flavoring Agents

**DOI:** 10.3390/dj10050085

**Published:** 2022-05-13

**Authors:** Christina P. Xu, Dominic L. Palazzolo, Giancarlo A. Cuadra

**Affiliations:** 1 Biology Department, Muhlenberg College, 2400 Chew Street, Allentown, PA 18104, USA; cxu@muhlenberg.edu; 2 Department of Physiology, DeBusk College of Ostheopathic Medicine, Lincoln Memorial University, 6965 Cumberland Gap Parkway, Harrogate, TN 37752, USA; dominic.palazzolo@lmunet.edu

**Keywords:** commensal bacteria, biofilms, electronic cigarettes, streptococci, E-liquids, toxicity, oral cavity, bactericidal, bacterial growth

## Abstract

**Background:** Vaping has become a global health concern. As research continues, more studies are beginning to question the relative safety of E-liquid flavoring additives. The oral cavity is the first site of exposure to E-liquid aerosol, making it critical for investigation. Because of the importance of commensal bacterial biofilms for oral health, we sought to explore the effects of E-liquids ± flavors on the formation and growth of single- and multi-species biofilms and to investigate the mechanism of inhibition. **Methods:** Quantitative and confocal biofilm analysis, death curves, and colony-forming units (CFU) were evaluated with flavorless and flavored (tobacco, menthol, cinnamon, strawberry, blueberry) E-liquids using four strains of oral commensal bacteria (*Streptococcus gordonii*, *Streptococcus intermedius*, *Streptococcus mitis,* and *Streptococcus oralis*). **Results:** All flavoring agents show a dose-dependent inhibition in the growth of single-species and multi-species biofilms. Furthermore, CFUs, death curves, and light microscopy show that flavoring agents have a bactericidal mode of inhibition on the growth of these oral streptococci. **Conclusions:** These results show that flavored, rather than unflavored, E-liquids are more detrimental to biofilm formation and growth of oral commensal bacteria. Consequently, E-liquid flavorings agents could pose risks to the oral microenvironment, and by extension, to systemic health.

## 1. Introduction

Electronic cigarettes (ECIGs), commonly known as vaping devices, or electronic nicotine delivery system (ENDS), were originally developed in China in 2003 and marketed towards those seeking to cease smoking [1]. As tobacco usage rates have decreased, ECIG usage rates have increased significantly, especially among adolescents [2]. ENDS are handheld devices that come in a variety of constructs, such as E-cigars, E-pipes, and E-hookahs [3]. Each device contains a rechargeable battery, a heating element with a resistance coil, and an atomizer tank or removable cartridge (e.g., pods) containing E-liquid. The E-liquid typically contains a mixture of nicotine, a myriad of flavoring agents, and the humectants vegetable glycerin (VG) and propylene glycol (PG). When the ENDS is actuated, the resistance coil is heated, and the surrounding E-liquid mixture is aerosolized. The user inhales the aerosol (i.e., vaping) in the manner of conventional cigarette smoke [4].

Many conventional smokers who want to quit have substituted traditional smoking with vaping to satisfy their nicotine urges. Smoke from conventional cigarettes is primarily hazardous because of the tar generated from combusting tobacco. Tar contains thousands of chemicals, many of which are known to be carcinogenic [1,5]. Smoke also contains nicotine, the addictive component of cigarettes. E-liquids similarly contain nicotine, but without tar. Anecdotally, many believe that vaping is a safer alternative to cigarette smoking because the nicotine addiction can still be satisfied without the perils of tar. Consequently, this harm-reduction approach to vaping is viewed by current smokers to be a useful smoking cessation option [6,7,8]. As ECIGs have become increasingly popular, they have also caught the attention of the media and health organizations, including the Office of the Surgeon General, to raise awareness about this trend which is now a public health concern.

E-liquids contain nicotine ranging from 0 to 87 mg/mL [9]. The introduction of flavoring compounds to E-liquids, which mimic fruits, desserts, candy, and beverages, has increased the usage of ECIGs in the past few years, especially among adolescents [2]. Adolescents and teenagers are generally naïve to smoking or vaping, and tend to be more vulnerable and willing to experiment with ECIGs. Thus, if these naïve individuals wish to experience new taste sensations of E-liquid flavors, they may unwittingly fall prey to nicotine addiction. E-liquid components are commonly available online. This not only facilitates the preparation of E-liquids at home, but may allow under-age individuals to circumvent purchasing ready-made E-liquids at the local vape shop. This new phenomenon called “Do-It-Yourself Vape Juice” allows for adolescents to experiment with a variety of different flavor profiles [10]. For adolescents, these home-made products have proven to be very enticing and addictive, with more accessible options and appealing flavors including, but not limited to, bubblegum, tutti-frutti, and blueberry. Therefore, flavored E-liquids are the most used nicotine product among this age group. For example, in 2020, approximately 5% of middle schoolers and 20% of high schoolers reported using these products in the last 30 days [2]. The Truth Initiative [11] communicated that 32.7% of US high school students (aged 14–18) have used vaping products, making youth tobacco usage a forefront epidemic. It is paradoxical that vaping, which started as a promising smoke-cessation option, has also become an alternative means by which E-liquid and tobacco companies take advantage of naïve individuals–especially adolescents–addicting them to nicotine and profiting in the process. 

Many studies have been conducted regarding the effects of vaping products on lung tissue and airway epithelial cells, arguing that these products have significant effects on respiratory health [12,13]. Studies have shown that flavorings such as strawberry, cinnamon, and menthol added in vaping products on top of the base humectants of PG and VG contribute to the detrimental effects on pulmonary tissues and cells such as cytokine production and cell death [14,15]. Furthermore, studies in human lung and airway epithelial cells, in the presence of ECIG aerosol and E-liquid diluted in media, result in damage to DNA, decrease in antimicrobial activity, increase in levels of cellular stress, decrease in cell proliferation, and other stress-induced detrimental effects [16,17,18,19]. These in vitro findings suggest that E-liquids induce toxic effects. A high incidence of E-cigarette/Vaping Associated Lung Injury (EVALI) has been reported [20,21]. EVALI is characterized by respiratory symptoms such as sudden lung infections, shortness of breath, cough, chest pain, fever, and chills [22]. While the effects of E-liquids on airway are under investigation, there are very few studies on the effects of E-liquids on the oral microenvironment (i.e., microbiota and underlining epithelium). 

The oral cavity contains the second highest concentration of bacteria in humans, with over 700 species present [23], and is home to pathogenic and commensal bacteria [24]. Initially, commensal bacteria interact with each other to form biofilm communities on tooth surfaces [25,26]. These biofilms are often extremely dense when growing on hard surfaces. Commensal species, mainly streptococci, play a role in preventing many oral diseases such as gingivitis, caries, and periodontal disease [27]. Studies have shown that commensal oral streptococci contribute to oral homeostasis through factors such as pH stability, while slowing growth of pathogenic species [28,29,30]. For example, a previous study from our group shows that commensals *Streptococcus gordonii* and *Streptococcus intermedius* reduce invasion of the oral pathogen *Porphyromonas gingivalis* into oral epithelial cells [31]. When this microbiome is destabilized by outside stressors such as cigarette smoke, the oral cavity becomes more susceptible to inflammation and infection, leading to diseases such as gingivitis and periodontitis [32,33]. Since the oral cavity is the first anatomical site to be affected by the exposure of cigarette smoke or ECIG aerosol, oral bacteria will likely be affected from this exposure. Previous studies by our group explored the effects of cigarette smoke, E-liquids (±flavors), and their aerosols with and without the addition of nicotine. Results suggest that ECIG-generated aerosols have less detrimental effects on oral commensal streptococci in comparison to conventional cigarette smoke [34,35]. The most recent study tested the effects of flavoring compounds in E-liquids on the growth of streptococci, in planktonic mode rather than biofilms, concluding that flavored E-liquids are more detrimental to the growth of these bacteria in comparison to the unflavored controls [36]. Other studies have shown that the normal antimicrobial properties of saliva can be compromised in a user of ECIGs, resulting in decreased levels of oral lysozyme and lactoferrin [37]. Another study demonstrated a shift in the composition of the oral microbiota as a result of vaping [38]. In addition, alterations in the beta-diversity (i.e., number of species and abundance of each) of the oral microbiome, such as higher levels of *Porphyromonas* and *Veillonella*, increase the risk of infection [39]. As a result of alterations in oral microbial communities’ beta-diversity, there is a risk for predominance of pathogenic species (i.e., dysbiosis) and impaired homeostasis, which can contribute to oral diseases [40], and may ultimately lead to more serious systemic complications. 

In this study, we evaluate the effects of various E-liquid flavorings on biofilm formation and toxicity of four oral commensal bacterial species: *Streptococcus gordonii*, *Streptococcus intermedius*, *Streptococcus mitis*, and *Streptococcus oralis*. We aim to test the effects of five commercially available E-liquid flavorings on oral biofilm formation and growth in an in vitro model system. A complete description of these flavors has previously been documented [36]. The aims of this study are two-fold: (i) to determine the effects of flavoring compounds on the formation and growth of single-species and multi-species oral commensal bacterial biofilms, and (ii) to explore the mechanistic mode of inhibition of E-liquid flavorings on oral bacteria. We hypothesize that exposure to E-liquid flavorings alters biofilm formation and growth. Since commensal oral biofilms are naturally occurring in the mouth, disruption of such communities could impact oral health. It is important to note that oral health is not isolated from the rest of the body. Consequently, it is critical to understand the ways in which vaping can impact the oral cavity, and therefore ultimately influence systemic health.

## 2. Materials and Methods

### 2.1. Reagents and Supplies

Reagents and supplies for this study were purchased from Fisher Scientific (Hampton, NH, USA) unless otherwise explicitly noted. 

### 2.2. Bacterial Strains

The bacterial strains tested were *S. gordonii* DL1, *S. mitis* UF2, *S. intermedius* 0809, and *S. oralis* SK139. These strains were originally donated by Dr. Robert Burne at the University of Florida, College of Dentistry in Gainesville, Florida, United States and have been routinely grown and passaged in our laboratory. These strains were grown in brain heart infusion (BHI) media with a supplemental addition of 5 μg/mL porcine hemin, and on BHI agar at 37 °C, 5% CO_2_ as previously described [31,36]. All bacterial stocks were stored in a −80 °C freezer and, prior to experiments, purity of individual strains was routinely confirmed by Gram staining and light microscopy. In addition, 16S rRNA sequencing (Genewiz, South Plainfield, NJ, USA) was performed to ensure the purity of each species. 

### 2.3. Stock E-Liquid

In this study, E-liquid preparations followed a strict protocol, allowing us to know the exact composition of each batch. A common problem with commercially prepared E-liquids is that they are proprietary, and therefore the exact composition is unknown in most cases. Furthermore, commercially prepared E-liquids are known to be highly variable from batch to batch [41]. Consequently, the base flavorless E-liquid solution was prepared with a 1:1 *v*/*v* mixture of PG and VG, obtained from Liquid Nicotine Wholesalers (Phoenix, AZ, USA), and spiked with 20 mg/mL of (S)-(-)-nicotine, 99% (Alpha Aesar, Tewksbury, MA, USA). Stock flavors were added to a final concentration of 25% (*v*/*v*) in the base flavorless E-liquid, which included tobacco, menthol, cinnamon, strawberry, and blueberry, as indicated in Table 1. These five flavors, also obtained from Liquid Nicotine Wholesalers (Phoenix, AZ, USA), were chosen because tobacco and menthol are commonly used among individuals trying to quit smoking, and cinnamon, strawberry, and blueberry are popular flavors among adolescents. As shown in Table 1, E-liquid solutions were made with concentrations of 0 or 25% stock flavoring and subsequently diluted to a final working concentration of 5%, 3%, and 1% in BHI broth for further experimentation. 

### 2.4. Saliva Preparation

Saliva donations were collected from five different participants at Muhlenberg College under IRB approval code Cuadra_S19_18. Following previously published saliva donation protocols [42,43], all participants were required to fit donation criteria: (i) non-smokers or vapers, (ii) no current antibiotic treatment, (iii) no food or drinks (except water) two hours prior to donation, and (iv) healthy status at the time of donation excluding any allergies. Raw saliva donations were stored at −20 °C. Raw saliva samples from the five donors were pooled and mixed on ice. Dithiothreitol was added to a final concentration of 2.5 mM to reduce disulfide bonds, stirring on ice for 10 to 15 min. All reduced saliva was then diluted 1:4 in distilled water. Diluted saliva was centrifuged at 14,000× *g* for 30 min, vacuumed through a 0.45 μm filter, and collected in a sterile container. All processed saliva was aliquoted into 25 mL tubes and stored indefinitely at −20 °C or at 4 °C for up to a week or before use.

### 2.5. Biofilm Assays and Confocal Analysis

One hundred microliters of processed saliva were added to each well of a 96-well plate for 48 h at 4 °C to generate a pellicle. All bacterial strains were streaked and cultured on BHI agar overnight at 37 °C and 5% CO_2_ and inoculated into BHI broth to grow batch cultures. After overnight incubation, batch cultures were adjusted to an optical density (OD) = 1.0 at 595 nm wavelength, approximately one billion bacteria/mL in the culture. These samples were diluted in 1:4 in BHI broth. Saliva was removed from wells and the existing pellicles were washed once with 100 µL phosphate buffered saline (PBS). Each species of diluted bacteria was subsequently added to saliva-treated 96-well plates in a final volume of 100 µL per well. Bacteria were incubated at 37 °C and 5% CO_2_ for one hour, allowing for adherence of streptococci species to the pellicle generated by the processed saliva. Excess bacterial cells were washed out twice with 100 µL/well PBS. Then, 100 µL of 50% BHI (control) or 50% BHI containing 5% hydrogen peroxide or 5%, 3%, or 1% concentrations of E-liquids ± 25% flavors were added to each well (Table 1). Single-species biofilms were grown overnight at 37 °C and 5% CO_2_ and the crystal violet staining procedure was used to quantify the amount of biofilm biomass [44]. Briefly, all spent media were discarded, and wells were washed with distilled water. One hundred microliters of 5% crystal violet solution (diluted in PBS) were added into each well for 10 min to stain the biofilms. Once stained, the crystal violet solution was discarded, and each well was washed five times with 100 µL distilled water. To extract the crystal violet stain from the biofilms, 100 µL of 3% acetic acid diluted in distilled water was added to all wells and shaken in a plate shaker for 1 min at 400 rpm. Then, the 3% acetic acid with any extracted crystal violet was transferred onto a new and untreated 96-well plate. The amount of extracted crystal violet in 3% acetic acid serves as an index of biofilm biomass. Absorbance of each well was read at 595 nm wavelength with a μQuant monochromatic microplate reader equipped with KC4 software version 3.4 (MTX Lab Systems, Bradenton, FL, United States). This method was repeated for all four species separately.

To form multi-species biofilms, all streptococci species were grown separately in BHI broth and then standardized to OD = 1.0 at 595 nm. Each species was then mixed in a 1:1:1:1 ratio. This mixture was then diluted 1:4 in BHI broth. Biofilm formation with 50% BHI broth, with and without E-liquids ± flavors or hydrogen peroxide, followed the same procedure as single-species biofilms above. For confocal analysis, multi-species biofilms were grown in chamber-slides with 3% E-liquids ± 25% flavors as above. Biofilms were washed and the DNA was stained with 5 µM SYTO 59 in PBS for 30 min at room temperature in the dark. Excess stain was washed twice with PBS. Biofilms were imaged using a Carl Zeiss LSM880 laser scanning confocal microscope (Carl Zeiss Inc. White Plains, NY, USA) at 630× magnification with oil immersion using an excitation wavelength of 610 nm. Optical slicing was set at 1 µm and Z-stacks were acquired at slow speed and high resolution. Three-dimensional images were rendered using the ZEN 3.5 software (Carl Zeiss Inc., White Plains, NY, USA). The confocal microscope used in this study and the time allowed for its use were generously provided by the Biological Sciences Department in the College of Arts and Sciences at Lehigh University (Bethlehem, PA, USA). 

### 2.6. Colony Forming Unit Assay

Batch cultures of all bacteria were grown and adjusted to OD = 1.0 as above. One milliliter of bacterial suspension was transferred to microcentrifuge tubes and centrifuged at 10,000× *g* for 10 min, then washed once with 1 mL PBS. To assess the mechanism of growth inhibition, pellets were resuspended in either 100 µL PBS (control) or 100 µL of each of the undiluted stock E-liquid flavors. Bacteria were then incubated at 37 °C and 5% CO_2_ for 15 min to allow flavors to completely bathe the bacteria. This was followed by addition of 1 mL of PBS to all bacterial suspensions and centrifugation as above. To completely remove excess flavors, bacteria were washed twice with 1 mL PBS and carefully decanted to ensure no bacterial loss. To measure any potential lysis and loss of bacterial biomass, pellets were weighed before and after treatments. Bacteria were resuspended in 500 µL PBS, serially diluted 1:10, and plated on agar to assess colony formation. The undiluted suspensions of bacteria were stained with 5% crystal violet and observed under light microscopy at 1000× to determine cellular morphology. The microscope used was a Nikon Eclipse TE2000-U inverted microscope equipped with a Nikon Digital Sight DS-Fi1 camera and NIS Elements Imagine Software (Nikon Instruments Inc., Melvin, NY, USA).

### 2.7. Death Curves

Batch cultures of each bacterial species were grown as indicated above and subsequently adjusted to OD = 1.0. One milliliter of bacteria suspension was transferred to microcentrifuge tubes and centrifuged as above. All excess media were removed and bacteria were washed with 1 mL PBS, twice. Flavorless E-liquid or E-liquids plus 25% menthol or cinnamon flavors only were diluted separately in PBS, ranging from a 0% (control) to 5, 10, 15, 20, and 25% E-liquid ± flavors. Bacterial pellets were resuspended in 100 µL of PBS without and with E-liquids ± flavors at all concentrations. Mixtures were vortexed for 10 s to mix well. Bacterial suspensions were immediately serially diluted 1:10 and 40 µL were plated on BHI agar. Liquid was allowed to be absorbed in the agar for a few minutes and agar plates were incubated at 37 °C and 5% CO_2_ overnight. Colony-forming units (CFU) counts were collected after 24 h to determine the amount of bacterial viability after exposure to the different concentrations of E-liquid ± cinnamon or menthol flavorings. This method was repeated for all four species individually.

### 2.8. Statistical Analysis

For all quantitative experiments, means and standard errors of the mean (SEM) were calculated and analyzed. Statistical significance, when comparing flavorless and flavored E-liquid conditions to each other, was determined using one-way ANOVA. For comparisons between percentages of each E-liquid ± flavors in biofilm biomass and death curve assays, a two-way ANOVA was used. For both one-way and two-way ANOVAs, the Bonferroni post-hoc analysis was used to determine differences between treatment groups. Student *t*-test was used to determine significance between pellet weights before and after stock flavor treatments. Significance was established at *p* < 0.05.

## 3. Results

### 3.1. Biofilm Assays

**Figure 1:**
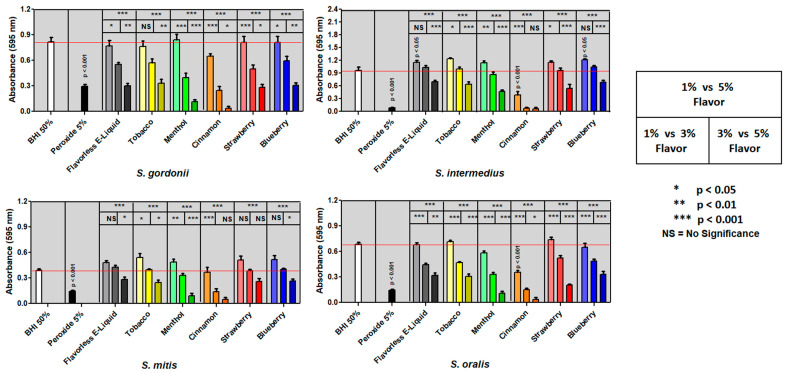
Dose-Dependent effect of E-liquids on Single-Species Biofilm Formation. Oral streptococci single-species biofilms biomass after treatment with 1, 3, and 5% E-liquids ± flavorings in 50% BHI. The color gradient for each E-liquid ± flavors ranges from light to dark indicating an increasing E-liquid percent. The given *p* values indicate significance between BHI 50% (positive control) and 5% peroxide (negative control) or BHI 50% and 1% E-liquid ± flavors using One-Way Anova. The red line indicates the average absorbance of the positive control. Three boxes above each E-liquid ± flavors indicate significance between them using Two-Way Anova. Each bar represents mean ± SE of absorbance (*n* = 10 to 24).

As expected, all four streptococci grow single-species biofilms with 50% BHI and are severely inhibited by the addition of 5% hydrogen peroxide (Figure 1). There is a significant dose-dependent biofilm inhibition of growth with increasing E-liquids ± flavors (Figure 1 and Table 2). E-liquids containing cinnamon inhibit biofilm formation and growth on all single-species tested more than any other flavor. For all streptococci, 5% E-liquid with cinnamon (and 3% E-liquid with cinnamon for *S. intermedius*) yields biofilm biomasses that approach the lower limit of detection. According to Table 1, 5% and 3% E-liquids with flavorings in BHI is equivalent to 1.25% and 0.75% final flavoring concentration in the broth, respectively. Menthol also significantly inhibits biofilm formation and growth in comparison to flavorless E-liquid (*p* < 0.05) (Figure 1 and Table 2). For *S. gordonii*, *S. oralis* and *S. mitis*, 5% E-liquid with menthol resulted in much lower biofilm growth compared to tobacco, strawberry, and blueberry (Figure 1). Interestingly, at 1% E-liquid ± flavors, the biofilm biomass was similar, or at times greater than that of BHI positive control. 

**Figure 2:**
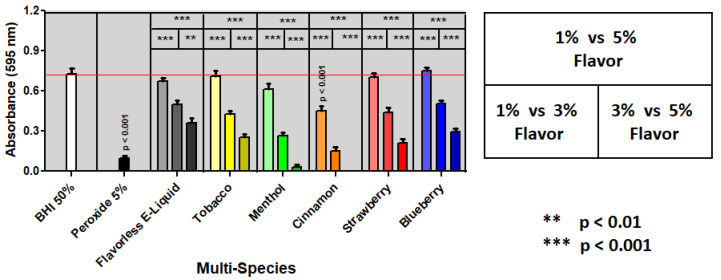
Dose-Dependent effect of E-liquids on Multi-Species (*S. gordonii*, *S. intermedius*, *S. mitis* and *S. oralis*) Biofilm Formation. Mixed streptococci (1:1:1:1) biofilms biomass after treatment with 1, 3, and 5% E-liquids ± flavorings in 50% BHI. The given *p* values indicate significance between BHI 50% (positive control) and 5% peroxide (negative control) or BHI 50% and 1% E-liquid ± flavors using One-Way Anova. The red line indicates the average absorbance of the positive control. The three boxes above each E-liquid ± flavors indicate significance between them using Two-Way Anova. Each bar represents mean ± SE of absorbance (*n* = 12 to 18).

Multi-species biofilms with all four species mixed in a 1:1:1:1 ratio were exposed to E-liquids ± flavors as previously described for single-species. The results collected (Figure 2) appear to match the trends found with single-species biofilms (Figure 1). Significant dose-dependent inhibitory effects are evident for all E-liquids ± flavors (Figure 2). The cinnamon flavoring results in significantly lower biofilm growth at 1% and 3%, but is undetectable at 5% (Figure 2). Menthol flavoring also strongly inhibits growth compared to tobacco, strawberry, and blueberry flavors (Figure 2). Our data suggest that E-liquids containing cinnamon and menthol flavorings most strongly hinder biofilm formation and growth of oral commensal bacteria in comparison to all other conditions tested. 

**Figure 3:**
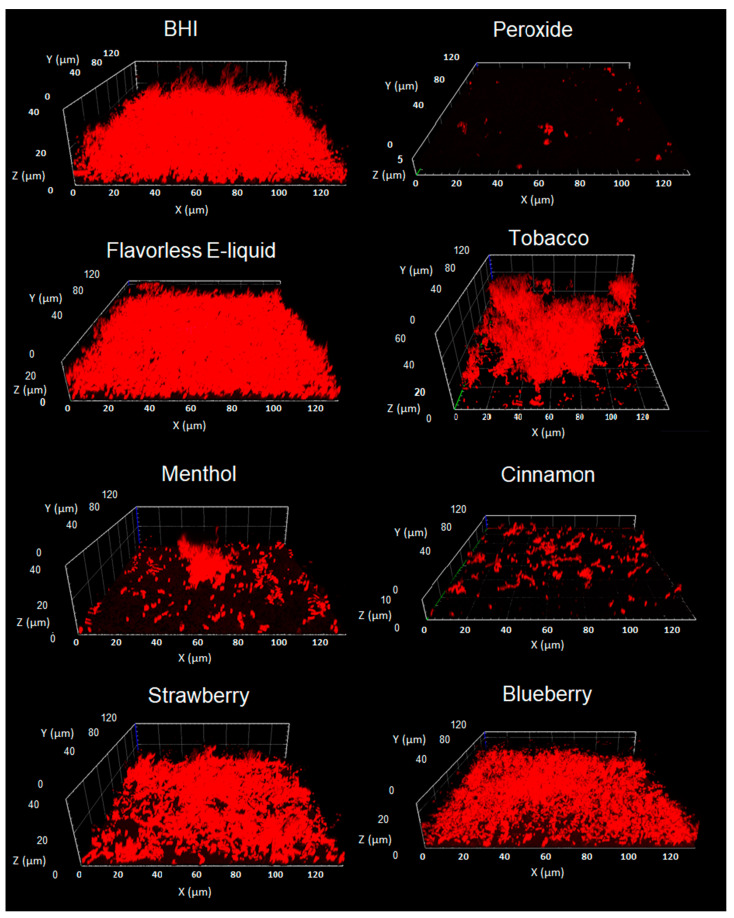
Effects of 3% E-liquids ± Flavors on Multi-Species (*S. gordonii*, *S. intermedius*, *S. mitis,* and *S. oralis*) Biofilm Formation. Confocal images of mixed streptococci (1:1:1:1) biofilms grown in 50% BHI with 3% E-liquids ± flavorings. The positive control is the 50% BHI alone. The negative control is the 5% Peroxide in 50% BHI. Biofilms were stained with SYTO 59 (red fluorescence). Each image is a typical representative of eight fields of view. Magnification = 630× Z-stacks were acquired at 1 µm thickness of optical slicing. Images are representative of eight different fields of view.

Confocal images of multi-species biofilms (shown on three dimensions; X, Y, and Z axes) treated with E-liquids ± flavors are depicted in Figure 3. Biofilms grown in 50% BHI (control) present a confluent community of roughly 20 µm in height (Z axis) with occasional bacterial extensions radiating above the 30 µm mark. In stark contrast, 5% peroxide in media yielded no significant biofilm formation showing only sparse bacterial aggregates. All four species are known to co-aggregate with each other [42,43,45,46,47,48], and most likely these aggregates represent any combinations of the four species adhered to each other and the saliva-coated surface. Biofilms grown with 3% flavorless E-liquid show a similar architecture and confluence as those grown in 50% BHI, albeit the height is slightly lower. Biofilms grown in media plus 3% E-liquid with tobacco flavoring display a different architecture, where communities grow in height to around 40 µm without achieving confluence. Biofilms grown with 3% E-liquid with cinnamon or menthol flavorings resemble the peroxide condition, but slightly enhanced bacterial aggregations. Finally, biofilms grown with 3% E-liquid with strawberry or blueberry flavorings resemble the architecture of the 50% BHI control and the flavorless E-liquid condition, but with slightly diminished confluence and biomass. These data support the biofilm biomass quantifications shown in Figure 2 and Table 2 indicating that both menthol and cinnamon flavors hinder biofilm formation of these commensal oral streptococci more than the other flavors tested.

### 3.2. Colony Forming Unit Assay

**Figure 4:**
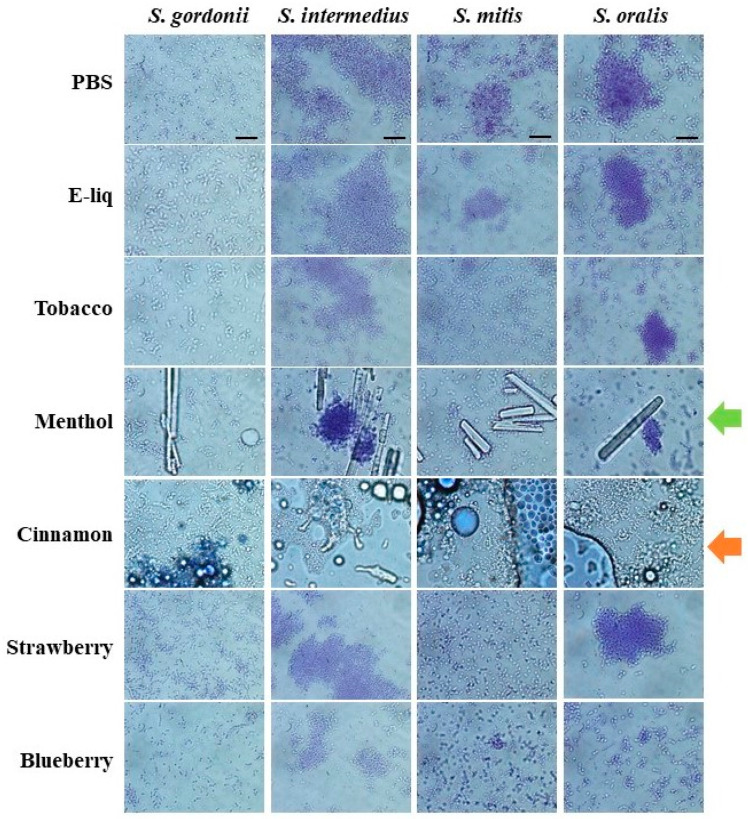
Streptococcal Morphology after E-liquid or stock flavor treatments. Light microscopy images were taken at 1000× magnification after treatment with 100 µL of PBS, flavorless E-liquid, or the five stock flavoring agents. The green arrow indicates menthol crystalline formation and the orange arrow points at the presence of a hydrophobic material associated with cinnamon. Black bars on top four images = 10 µm. Images are representative of four different fields of view.

After treating with undiluted stock flavoring agents or flavorless E-liquid and examining with light microscopy, all bacteria remain intact. For all four species, cells appear to be uncompromised and display similar morphologies when comparing controls to the treatments (Figure 4). In addition, Table 3 shows the growth, or lack thereof, of all species on agar after the same treatments. All four streptococci grow after treatment with flavorless E-liquid. Treatment with the tobacco flavoring results in reduced numbers of colonies. However, treatments with menthol, cinnamon, strawberry, and blueberry result in complete obliteration of growth, potentially indicating bacterial death (Table 3). These results suggest that out of all the flavors, tobacco is the least harmful for all four *spp.* as they can still grow, but to a lesser degree when compared to flavorless E-liquid. 

Based on Figure 1, Figure 2 and Figure 3, and Table 2, as well as our previous findings on planktonic inhibition [36], cinnamon and menthol appear to have the strongest inhibitory effects on biofilm formation compared to the other flavorings. Therefore, the remaining experiments will focus on the effects of cinnamon and menthol on all four bacteria.

### 3.3. Death Curves

**Figure 5:**
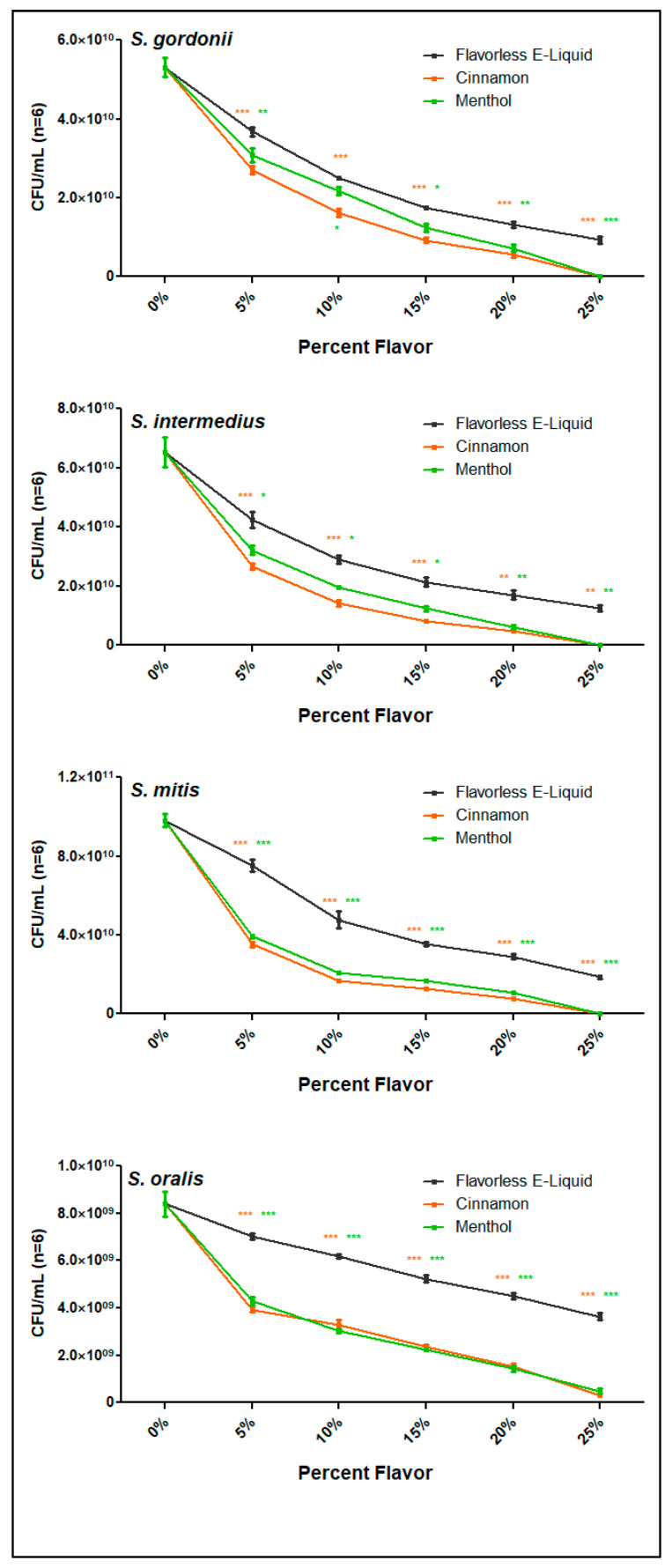
Death Curves of Streptococci after E-liquid ± menthol or cinnamon. Death curves performed with all four species of streptococci using E-liquid ± flavors from 0 to 25% in PBS. Each data point represents mean ± SEM (n = 6). Green stars and orange stars indicate significance between flavorless E-liquid and menthol and cinnamon, respectively. * *p* < 0.05, ** *p* < 0.01 and *** *p* < 0.001.

Figure 5 shows a significant dose-dependent decrease in bacterial viability after exposure to PBS containing 0 to 25% E-liquid with or without menthol or cinnamon flavors in 5% increments (*p* < 0.001) for all species. Additionally, E-liquids containing cinnamon or menthol flavors reduced the numbers of viable bacteria compared to flavorless E- liquid in all four species at every dose tested. For *S. oralis*, there was a striking decrease in bacterial viability with menthol and cinnamon, showing that this species is more susceptible to these flavoring agents than the other three. Altogether, our data demonstrate that these two flavoring agents have an obvious and overwhelming significant effect on the survival of all four oral streptococci tested in a dose-dependent manner.

### 3.4. Bacterial Mass Pre- and Post-Treatments

**Figure 6:**
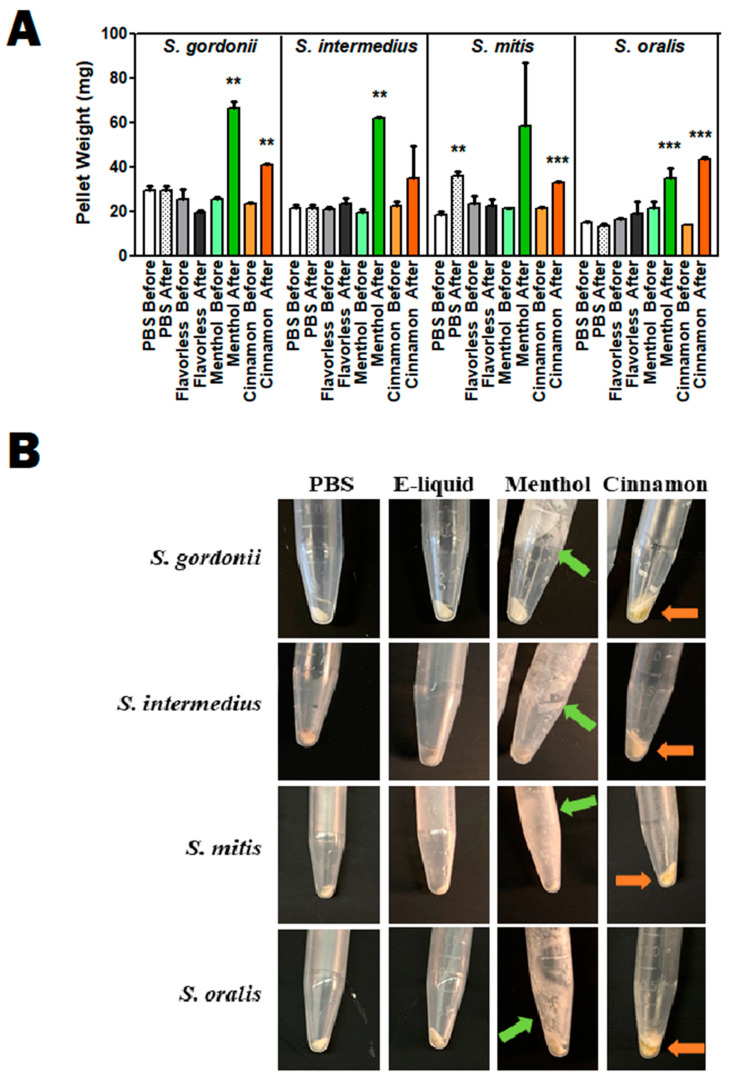
(**A**). Pellet Mass before and after E-liquid or stock flavor treatments. The weight of oral streptococci pellets was determined before and after treatment with 100 µL of PBS, or 100 µL of flavorless E-liquid, or cinnamon or menthol stock flavorings. Each bar represents mean ± SEM (*n* = 3). ** *p* < 0.01, *** *p* < 0.001. (**B**). Pellet size of representative centrifuge tubes. Bacteria were exposed to 100 µL of PBS, flavorless E-liquid, or cinnamon and menthol flavoring agents for 15 min. Bacteria were then washed and pelleted. Green arrows indicate precipitation of menthol crystals and orange arrows indicate the presence of a hydrophobic material associated with the cinnamon flavor.

Treatments with undiluted stock flavors show bacterial death (Table 3) and possibly bacterial lysis. However, light microscopy in Figure 4 reveals intact bacterial cells. To further evaluate the possibility of bacterial lysis upon stock flavoring treatments, bacteria were resuspended in these flavoring agents and samples (microcentrifuge tube + bacterial pellet) were weighed pre- and post-treatment. If lysis should occur, streptococcal cells would become cellular debris from the lysing activity and most of this cellular material would be removed with the supernatant. Consequently, the weight of the sample would be reduced post-treatment and the pellet would appear smaller. Figure 6A quantifies the weight of streptococcal samples before and after treatments with flavorless E-liquid, and undiluted stock cinnamon or menthol flavorings. There is little to no change in sample weights before and after PBS and E-liquid treatments, suggesting no bacterial lysis, indicating that the biomass for these conditions is unaltered. In the case of *S. mitis*, there is a modest increase of the sample weight, which could be a result of the remaining PBS in the tube. Removing all PBS volume without disturbing the bacterial pellet is technically challenging and could account for this increase. Unexpectedly, the weight of the menthol- and cinnamon-treated samples increases post-treatments (Figure 6A). Upon visual inspection (Figure 6B), menthol crystals are clearly evident in the centrifuge tubes, indicated by the green arrows, most likely contributing to this increase in weight. Similarly, an amber colored material associated with the stock cinnamon flavor appears to be trapped within the pellets, indicated by the orange arrows (Figure 6B), again adding to the final weight of this sample. In Figure 4 there are visible microscopic hydrophobic droplets among the bacteria in water-based PBS (orange arrow). In Figure 6B there is an amber colored material (orange arrows) that does not mix with the water-based PBS, hence not removed during the washing step. This most likely correlates with the microscopic hydrophobic droplets in Figure 4, which contain inherent organic materials from the stock cinnamon flavor and do not dissolve in aqueous solutions. Overall, Figure 6 demonstrates that treatments with stock menthol and cinnamon flavors do not decrease the actual biomass of the four streptococci tested, suggesting there is little to no bacteriolytic effect from these flavors, which support the results seen in the micrographs in Figure 4.

## 4. Discussion

Oral commensal biofilms are essential for homeostasis of the oral cavity. Considering the evidence that planktonic growth of oral commensal bacteria is negatively affected by E-liquid flavorings [36], in this study we sought to determine the impact of E-liquid flavorings on biofilm formation as single-species (Figure 1) or in multi-species communities of oral commensal bacteria (Figure 2 and Figure 3). Altogether, our work demonstrates that biofilm formation and growth are severely affected by E-liquids ± flavors in a dose-dependent fashion (Figure 1 and Figure 2; Table 2) and is supported by confocal analysis (Figure 3). The mixture of PG, VG, and nicotine alone are enough to produce this inhibitory effect. The addition of flavors may enhance such effect. On visual inspection, Figure 3 and Figure 4 demonstrate that, after treatments with E-liquids ± flavors, bacterial cells are still intact. However, Table 3 indicates that treatment with these E-liquids ± flavors (except tobacco) stops their growth. Interestingly, tobacco flavoring also induces a different biofilm architecture compared to control biofilms (Figure 3). At this time, there are no reports on the viability and biofilm architecture of oral bacteria, induced by tobacco flavoring, that would explain these findings; except to say that this flavoring alters the microbiology of these *spp.* From a clinical perspective, studies performed comparing the oral microbiome diversity sampled from smokers and vapers found a unique periodontal microbiome where relative concentrations of streptococci species, including *Streptococcus sanguinis*, *Streptococcus parasanguinis*, *Streptococcus anginosis*, and *S. gordonii* were altered in vivo [49]. This was also seen in experiments comparing relative mean abundance, with a net decrease in streptococci in ECIG users [49]. This is consistent with our findings, as streptococci species grown in biofilms exposed to E-liquids result in a significantly lower biomass.

Since cinnamon and menthol flavors cause the most significant effects, we narrowed the rest of the study to test just these two flavors. Figure 5 shows that cinnamon and menthol, compared to flavorless E-liquid, significantly inhibit bacterial growth. Figure 6 illustrates that the size and weight of the pellets after treatment with flavorless E-liquid or cinnamon or menthol do not decrease. Based on these findings, we propose flavoring agents to be bactericidal (i.e., bacteria present, but dead) but not bacteriolytic (i.e., bacterial remnants/cellular debris) on the four oral *spp.* tested in this study. 

The results of this study are consistent with previous findings from our group [34,35,36], as well as others [50,51,52] regarding vaping and the oral cavity, underlining the effects of a variety of E-liquids and their aerosols on oral streptococci. All data collected in this investigation extend the previous studies [36] while providing some mechanistic details. Specifically, there is a direct correlation between the increase in dosage of E-liquids ± flavors and the augmented inhibition of biofilm formation and growth of the *spp.* tested, even in the absence of flavoring agents. In particular, 3% and 5% E-liquids ± flavors significantly inhibited biofilm formation and growth, while the lowest percentage of 1% showed little to no statistically significant effect on the growth in comparison to the BHI control (Figure 1, Figure 2 and Figure 3; Table 2). Among the flavors tested, menthol and cinnamon have the highest effect on single- and multi-species biofilm formation and growth, as demonstrated by reduced biofilm biomass (Figure 1, Figure 2 and Figure 3; Table 2). These results are in line with previous data on the same four species of oral bacteria [34,35,36]. Interestingly, although there are no significant differences between single-species biofilms grown with strawberry flavored E-liquid compared to the flavorless E-liquid controls, there is a significant decrease in the amount of multi-species biofilm biomass for this condition (Table 2), suggesting that these species are more sensitive to the strawberry flavoring agent when residing in microbial communities. Of the five flavors tested, cinnamon and menthol are most consistent in biofilm growth inhibition across the four species alone or as a microbial community (Figure 1, Figure 2 and Figure 3; Table 2). This general trend is supported by the death curve experiments, where both cinnamon and menthol flavoring agents significantly reduced CFU counts in comparison to the flavorless E-liquid control (Table 3 and Figure 5). Additionally, the CFU assays show the mechanistic effects of the flavoring compounds, as light microscopy indicates that bacteria remain intact post-treatment (Figure 4), as well as the pellet biomass (Figure 6), indicating no bacteriolytic effects. 

Our group has previously shown the inhibitory effects of flavoring compounds, which are added to the flavorless E-liquids humectants: PG and VG. Studies regarding harmful flavoring chemicals when inhaled, started in the early 2000s, when workers in microwave popcorn factories were inhaling chemicals coming from butter flavoring, similar to flavoring compounds mimicking food flavors [53]. Diacetyl is the main ingredient in butter flavoring, and the same as those found in studies evaluating numerous E-liquid flavors [47]. Diacetyl and two closely related chemicals (2,3-pentanedione and acetoin) have been found in high concentrations in some flavoring agents, which may enhance inhibitory effects [53]. Moreover, creation of “Do-It-Yourself Vape Juice” among adolescents too young to purchase E-liquids adds additional risks since these home-made concoctions lack quality control assurances. Such practices greatly increase the percentage of flavoring compounds in the mixture, thereby amplifying the potential risk to overall health [54]. 

In this study, menthol and cinnamon flavorings are the most damaging to biofilm formation and growth of oral commensal streptococci. Treatments with stock cinnamon, followed by washes and centrifugation of bacteria, resulted in pellets with a hydrophobic amber material (Figure 6), which was also observed as droplets under light microscopy (Figure 4). Most likely, this material is trans-cinnamaldehyde which, according to the safety data sheet (SDS), is in liquid form at room temperature [55]. These droplets contain organic materials sequestered from the aqueous fraction of the stock cinnamon flavor in PBS, but remain unknown. Menthol treatments resulted in formation of crystallized materials left in the centrifuge tube after multiple washes, also observable under light microscopy. Undiluted stock menthol flavor (as with all flavors used in this study) is dissolved in propylene glycol [36], an organic solvent that prevents its crystallization at room temperature. According to the SDS, menthol crystallizes at 28 °C [56] and the PBS washes were conducted at room temperature (22 °C), which explains this phenomenon. Additionally, after decanting the PBS from the microcentrifuge tubes, trace volumes of PBS were inevitably left behind. Attempting to remove all PBS would result in partial loss of the bacterial pellet, thereby affecting its final weight. Unfortunately, this contributes to the variability of the mass within the microcentrifuge tubes (i.e., bacterial mass, hydrophobic materials, menthol crystals, and trace PBS), and could account for the significance noted pre and post PBS treatments for *S. mitis* (Figure 6A). Albeit, quantitative results in Figure 6A, and qualitative visual inspection of the pellets in Figure 6B demonstrate no loss of bacterial mass following treatments, negating a bacteriolytic effect.

The results of the bactericidal effects of menthol and cinnamon in this report correlate well with the antimicrobial properties shown by others [57,58,59,60,61,62]. For example, cinnamaldehyde has detrimental effects on the bacterial cell morphology and cell membrane integrity of *Escherichia coli* and *Staphylococcus aureus* [57], possibly explaining the major inhibitory effects observed in this study. A recent investigation by Silva et al. (2022) shows that essentials oils from the peppermint plant *Mentha piperita* yield a low minimal inhibitory concentration on Gram positives *Staphylococcus aureus* and *Listeria monocytogenes* [58]. Additional biochemical studies with cinnamon and menthol flavoring agents will help uncover the specific bactericidal mechanisms on oral streptococci.

Multi-species biofilms appear to be more susceptible to E-liquids with flavors, compared to single-species biofilms. As a matter of speculation, perhaps important interactions that normally occur among these four species, such as chemical communication (i.e., quorum sensing), are altered when exposed to E-liquids with flavors. For example, d-arabinose inhibits formation of *S. oralis*, *Fusobacterium nucleatum* and *P. gingivalis* multi-species biofilms of by abolishing the quorum sensing signaling molecule, autoinducer-2 [59]. Similarly, trans-cinnamaldehyde, a component of cinnamon flavor, inhibited *Streptococcus mutans* sucrose-dependent biofilm formation [60]. Due to the complex nature of polymicrobial interactions, further studies on oral biofilms with specific flavoring compounds are required to unravel the effects of E-liquids ± flavors on the oral microbiota. 

This study was limited to four oral streptococci species that are primary colonizers in the oral microbiota and were chosen for their role in the development of oral biofilms (e.g., dental plaque) [61]. Human saliva was used to simulate the acquired pellicle, which serves as the mechanism to initiate bacterial adherence and biofilm growth conditions in vitro [62,63,64,65]. However, despite these in vitro efforts, there are other limitations that are challenging to recreate, including: (i) the overwhelming number oral bacterial species in the mouth, (ii) the overall relative abundance of each species (iii) the interactions among commensal species, (iv) the putative presence of pathogenic species, (v) the flow of saliva over time, and (vi) the topography of vaping sessions (i.e., personal preference of puff duration, inter-puff duration, puff flow rate), to name a few. Additionally, E-liquids in this study were either diluted in BHI or used as undiluted stock flavors and directly added onto the bacteria without aerosolization. The process of aerosolization has the potential to alter the chemical nature of E-liquids [66]. Our previous findings have shown that the overall chemical load of vaporized E-liquid is typically lower in comparison to E-liquid diluted in the media [36]. This means that equal volumes of aerosol and E-liquid result in a greater effect of the latter on the growth of oral streptococci, but does not take into account the uniformity of aerosol generated based on its constituents. Nonetheless, the information gained from this study identifies potential risks associated with the more realistic effect of ECIG-generated aerosol on oral commensal bacterial communities. The volume of the oral cavity is roughly 230 cm^3^ according to Kaufman and Farahmand [67]. This is a significantly larger volume than the vessels used in this study, which means that the effect of E-liquids ± flavors presented is amplified. Furthermore, only a fraction of the total aerosol inhaled into the oral cavity is dissolved into the saliva. Therefore, most of the aerosol ends up in the respiratory tract, thus limiting the chemical load in the mouth [68]. Another fact to consider is that the continuous flow of saliva across the oral cavity further lowers the chemical load on the oral microbiota as old saliva is washed away and replaced by new saliva. Lastly, this study does not take into consideration the possible interactions between the flavoring agents and numerous other trace constituents known to exist in E-liquids and their aerosols. For example, previous studies have shown the presence of trace metals in aerosolized E-liquids [69,70] that could potentially impact the normal growth of biofilms. To date, there are no documented effects of any of these trace constituents on the growth of oral commensal bacteria. 

Experiments are currently being designed to investigate the effects of E-liquids on polymicrobial biofilms under similar conditions, containing both commensal and pathogenic species such as *P. gingivalis*, *F. nucleatum,* and *Aggregatibacter actinomycetemcomitans*. With the addition of these three species, it will be possible to evaluate how commensal and pathogenic microbes interact after exposures to E-liquids ± flavors. Such studies may provide a clearer understanding into interspecies interactions, as well as change in abundance and composition of *spp.* involved. For example, signaling molecules involved in bacterial communication, physical coaggregation between species, and metabolites such as adenine-derived molecules will be explored after E-liquid ± flavors exposure. Importantly, polymicrobial communities treated with E-liquids ± flavors could result in dysbiosis, leading to oral diseases. Moreover, future studies will employ more realistic conditions such as a continuous supply of human saliva to mimic biofilm formation in the oral cavity with intermittent addition of flavored E-liquid compounds. Other studies being considered include in-depth chemical analyses, using techniques such as gas chromatography, high performance liquid chromatography, and nuclear magnetic resonance spectroscopy, to determine which compounds are found most frequently in these flavoring agents and which compounds contribute to the toxicity and decreased growth patterns found in the oral microbiota. 

## 5. Conclusions

This study has shown that E-liquids containing flavoring compounds at high concentrations have significant inhibitory effects on biofilm formation and growth of oral commensal streptococci, both as a single-species and as a multi-species community. Mechanistically, the flavoring compounds exhibit a bactericidal effect, killing the streptococci, but keeping their morphology intact. These findings promote further understanding of the effects of E-liquids on the implications of oral health. Dysbiosis of the oral microbiota has been implicated in dental caries, periodontitis, gingivitis, and many other oral diseases [28,71]. The diverse and complex oral microbiota may be severely impacted by altering the abundance of pathogenic bacteria, at the expense of the commensal microorganism, resulting in detriment to the rest of the oral microenvironment. Commensals are extremely important in maintaining homeostasis of the oral microenvironment, and variations to the oral microbiome may prove to have detrimental effects beyond the oral cavity leading to systemic pathological processes [72]. Exploring the effects of vaping in oral biology deserves more attention as oral health impacts, and is greatly correlated to, systemic health. 

The results of these experiments contribute significantly to the understanding of vaping effects oral health by demonstrating that E-liquids ± flavors are detrimental to the in vitro growth of oral commensal bacterial biofilms, following a bactericidal mechanism. These oral streptococci are primary colonizers in dental plaque and biofilm communities in the oral cavity, and their growth is crucial to the homeostasis of this anatomical site. Flavoring agents of E-liquids may pose a risk to the health of the oral microenvironment, and thereby systemic health.

## Figures and Tables

**Table 1:** Final percentage of flavors in the working BHI broth.

	E-Liquid Constituents				Percent Stock E-Liquid added to BHI *		
Stock E-Liquid	Propylene Glycol	Vegetable Glycerine	Stock Flavor	Nicotine (mg/mL)	5%	3%	1%
Flavorless	50%	50%	0%	20	0%	0%	0%
Flavored	37.5%	37.5%	25%	20	1.25%	0.75%	0.25%

* Final percentage flavoring in the working BHI broth after the addition of 5, 3, and 1% stock E-liquids.

**Table 2:** Comparison of Absorbance Readings of Flavorless E-liquid vs. each of the Flavored E-liquids.

*S. gordonii*	Flavorless	Tobacco	Menthol	Cinnamon	Strawberry	Blueberry
1% E-liquid	0.77 ± 0.07 *	0.76 ± 0.07	0.84 ± 0.07	0.65 ± 0.03	0.81 ± 0.07	0.81 ± 0.07
3% E-liquid	0.55 ± 0.03	0.57 ± 0.05	0.40 ± 0.05	0.25 ± 0.05 ***p* < 0.001**	0.50 ± 0.05	0.60 ± 0.05
5% E-liquid	0.30 ± 0.03	0.33 ± 0.04	0.12 ± 0.02 ***p* < 0.01**	0.04 ± 0.03 ***p* < 0.001**	0.29 ± 0.03	0.31 ± 0.03
***S. intermedius***	**Flavorless**	**Tobacco**	**Menthol**	**Cinnamon**	**Strawberry**	**Blueberry**
1% E-liquid	1.16 ± 0.04	1.23 ± 0.03	1.15 ± 0.04	0.38 ± 0.08 ***p* < 0.001**	1.16 ± 0.04	1.21 ± 0.03
3% E-liquid	1.04 ± 0.04	1.00 ± 0.04	0.89 ± 0.06	0.07 ± 0.03 ***p* < 0.001**	0.96 ± 0.06	1.05 ± 0.03
5% E-liquid	0.70 ± 0.04	0.63 ± 0.07	0.46 ± 0.04	0.07 ± 0.04 ***p* < 0.001**	0.55 ± 0.10	0.69 ± 0.05
***S. mitis***	**Flavorless**	**Tobacco**	**Menthol**	**Cinnamon**	**Strawberry**	**Blueberry**
1% E-liquid	0.48 ± 0.03	0.54 ± 0.05	0.49 ± 0.03	0.37 ± 0.06	0.54 ± 0.05	0.52 ± 0.05
3% E-liquid	0.43 ± 0.03	0.39 ± 0.01	0..33 ± 0.02 ***p* < 0.05**	0.14 ± 0.03 ***p* < 0.001**	0.39 ± 0.02	0.40 ± 0.02
5% E-liquid	0.28 ± 0.03	0.25 ± 0.03	0.09 ± 0.02 ***p* < 0.001**	0.05 ± 0.03 ***p* < 0.001**	0.26 ± 0.03	0.27 ± 0.02
***S. oralis***	**Flavorless**	**Tobacco**	**Menthol**	**Cinnamon**	**Strawberry**	**Blueberry**
1% E-liquid	0.68 ± 0.02	0.72 ± 0.02	0.58 ± 0.02	0.35 ± 0.03 ***p* < 0.001**	0.74 ± 0.03	0.65 ± 0.05
3% E-liquid	0.44 ± 0.02	0.47± 0.02	0.33 ± 0.02 ***p* < 0.01**	0.15 ± 0.02 ***p* < 0.001**	0.52 ± 0.03	0.49 ± 0.02
5% E-liquid	0.32 ± 0.03	0.30 ± 0.03	0.11 ± 0.03 ***p* < 0.001**	0.04 ± 0.03 ***p* < 0.001**	0.20 ± 0.01	0.33 ± 0.04
**Mixed Species ^#^**	**Flavorless**	**Tobacco**	**Menthol**	**Cinnamon**	**Strawberry**	**Blueberry**
1% E-liquid	0.67 ± 0.03	0.71 ± 0.04	0.61 ± 0.04	0.45 ± 0.04 ***p* < 0.001**	0.70 ± 0.03	0.75 ± 0.02
3% E-liquid	0.50 ± 0.03	0.43 ± 0.02	0.27 ± 0.03 ***p* < 0.001**	0.15 ± 0.03 ***p* < 0.001**	0.44 ± 0.03	0.51 ± 0.03
5% E-liquid	0.36 ± 0.04	0.25 ± 0.03	0.31 ± 0.02 ***p* < 0.001**	−0.14 ± 0.01 ***p* < 0.001**	0.21 ± 0.03 ***p* < 0.01**	0.30 ± 0.02

* All values expressed as mean ± SEM of absorbance (*n* = 10 to 24). ^#^ *S. gordonii*, *S. intermedius*, *S. mitis*, and *S. oralis* mixed at a 1:1:1:1 ratio.

**Table 3:** Colony Forming Unit Assay.

	*S. gordonii*	*S. intermedius*	*S. mitis*	*S. oralis*
PBS	+++	+++	+++	+++
Flavorless E-liquid	++	+++	+++	++
Tobacco	+	+	++	++
Menthol	−	−	−	−
Cinnamon	−	−	−	−
Strawberry	−	−	−	−
Blueberry	−	−	−	−

Each assay consisted of n = 4 and the amount of CFUs was visually ranked as follows: +++ bacterial lawns; ++ too many to count; + countable colonies; − growth was not detected.

## Data Availability

The contributions presented in the study are included in the publication. Any further questions or inquiries can be directed to the corresponding author.
